# Absent mindfulness: mediation analyses of the relationship between adverse childhood experiences and disordered eating among young adults

**DOI:** 10.3389/frcha.2023.1148273

**Published:** 2023-05-26

**Authors:** Michael F. Royer, Kelly Cosgrove, Christopher Wharton

**Affiliations:** College of Health Solutions, Arizona State University, Phoenix, AZ, United States

**Keywords:** disordered eating, young adults, mediation, adverse childhood experiences, mindfulness

## Abstract

Recent evidence has indicated that adverse childhood experiences (ACEs) involving abuse, neglect, and other potentially traumatic occurrences are predictive of disordered eating among young adults. Previous findings have suggested that ACEs and disordered eating were both inversely related to mindfulness. No known studies have examined the extent to which mindfulness mediates the link between ACEs and disordered eating. This study was conducted among a sample of 144 young adults in the U.S. between the ages of 18 and 26 years. Primary study variables included ACEs, mindfulness, and disordered eating. Univariate and multivariable regression analyses assessed the link between ACEs and disordered eating. Mediation analyses examined whether mindfulness mediated the link between ACEs and disordered eating. Multivariable analyses statistically adjusted for the covariates of age, sex, race/ethnicity, and income. Findings suggested ACEs were inversely related to mindfulness (*B* = −0.04, SE = 0.01; 95% CI = −0.07, −0.01; *p* < 0.05), mindfulness was inversely related to disordered eating (*B* = −1.27, SE = 0.23; 95% CI = −1.74, −0.80; *p* < 0.0001), and ACEs were positively related to disordered eating before (*B* = 0.17, SE = 0.04; 95% CI = 0.09, 0.26; *p* = 0.0001) and after (*B* = 0.13, SE = 0.04; 95% CI = 0.05, 0.21; *p* = 0.002) adjusting for mindfulness. Mediation analysis results indicated that the link between ACEs and disordered eating was significantly mediated by mindfulness (*B* = 0.05, SE = 0.02; 95% CI = 0.01, 0.09; *p* < 0.05). Evidence produced in this study confirmed existing findings concerning the positive association between ACEs and disordered eating among young adults, and these outcomes helped fill a knowledge gap regarding whether mindfulness mediates the link between ACEs and disordered eating. Future intervention studies should identify health-enhancing mindfulness approaches and then test whether the adoption of mindfulness practices can alleviate and prevent disordered eating among young adults with high ACEs.

## 1. Introduction

### 1.1. Background for adverse childhood experiences and disordered eating

Adverse childhood experiences (ACEs) are characterized by abuse (physical, emotional, sexual), neglect (physical, emotional), and household dysfunction (witnessing abuse, divorce, incarceration, substance abuse, mental illness) occurring from the ages of 0–17 years old (1). ACEs are a major risk factor for the emergence of risky behaviors and poor health conditions in adulthood (2). Among adults with high ACEs, the pairing of persistently high stress levels with unhealthy coping mechanisms further increases the risk for chronic disease (3).

During the 21st century, there have been declines in a variety of ACEs including childhood poverty, exposure to domestic violence, parental divorce, physical abuse, emotional abuse, and sexual abuse in comparison to the late 20th century (4). In contrast, the ACEs that have consistently increased in prevalence since the 20th century have been parental alcohol and drug abuse (4). An examination of data derived from the 2016 National Survey of Children's Health (NSCH) revealed that, among 45,287 U.S. children aged 0–17 years, almost half (45%) had experienced at least one ACE while one in 10 encountered three or more ACEs (5).

Widespread disparities in ACEs exist across sociocultural groups in the United States (6, 7). ACEs are most common among individuals who are low-income (8); female (7); and those who are Black, Indigenous, and people of color (6). Recent reports have highlighted how ACEs were higher among Black (61%) and Hispanic (51%) children than White (40%) and Asian (23%) children (5). Adults with four or more ACEs have significantly worse health outcomes than those with no ACEs (9). Chronic diseases that are associated with high ACEs include obesity (10), asthma (11), heart disease (12), cancer (13), and stroke (12). High ACEs also increase the odds of mental illness and associated symptoms in adulthood including anxiety (14), depression (15), and suicidal behavior (16). Certain unhealthy behaviors contributing to the development of disease among adults with high ACEs involve physical inactivity (17), illicit drug use (18), binge drinking (19), and disordered eating (20).

Disordered eating is characterized by eating behaviors typically exhibited by individuals with eating disorder symptoms in the absence of a clinical diagnosis (21). Specific disordered eating behaviors include restraint, eating concern, weight concern, and shape concern (22). Restraint involves an avoidance of eating, eating concern pertains to a fixation with food, weight concern refers to a preoccupation with one's own bodyweight, and shape concern involves worries about one's own body composition. Since healthy eating is vital for human wellbeing (23), the harmful impact of certain types of disordered eating behaviors pose a threat to both physical health (24) and mental health (25), as disordered eating has been shown to increase the odds of obesity (26), type 2 diabetes (27), and mental health problems including depression (28), and suicidal behaviors (29).

### 1.2. Adverse childhood experiences and disordered eating among young adults

A positive association between ACEs and disordered eating has been detected in several samples of college students (30–33), while only two known studies have confirmed these findings among young adult samples that are more representative of the general U.S. population (20, 34). Outcomes from recent research among a diverse sample of young adults in the midwestern U.S. (*n* = 1,647) highlighted how both emotional abuse (RR = 1.4, 95% = 1.1, 1.8) and emotional neglect (RR = 1.4, 95% = 1.2, 1.8) in childhood were connected to a greater risk of disordered eating during adulthood (34). Findings from a separate study among another diverse sample of young adults in the midwestern U.S. (*n* = 1,440) suggested that young adults who experienced household dysfunction (*B* = 1.22, 95% CI = 1.02, 1.47) or household dysfunction and abuse (*B* = 1.89, 95% CI = 1.39, 2.57) during childhood were also at an increased risk of disordered eating (20).

Additional research findings for the relationship between adverse childhood experiences and disordered eating in young adult males have shown that a higher prevalence of those with an adverse family background reported disordered eating (*χ*^2^ = 9.11, DF = 1; *p* = 0.003) compared to those without an adverse family background, while no differences in disordered eating were detected between victims and non-victims of physical abuse and sexual abuse (30). Contrarily, a study among young adult males determined that physical abuse during childhood was correlated with disordered eating in adulthood (*r* = 0.26, *p* < 0.01). In the same study, physical neglect during childhood was also found to be correlated with disordered eating (*r* = 0.35, *p* < 0.02). Mediators were explored in this study, but it was found that neither anxiety nor alexithymia were significant mediators within the relationship between ACEs and disordered eating (31).

Additional outcomes from research that examined the relationship between ACEs and disordered eating among young adults revealed how childhood experiences of sexual abuse [*t* (1,153) = 2.31, *p* = 0.02] and neglect [*t* (1,153) = 6.26, *p* < 0.001] were both associated with disordered eating in adulthood. However, results from a mediation analysis in the same study reported that anxiety mediated the relationship between sexual abuse and disordered eating, as it was no longer significant after accounting for anxiety during adulthood [*t* (1,092) = 0.98, *p *= 0.34] (32). Moreover, other mediation-related findings have indicated that experiences of emotional abuse are linked to an increased likelihood of disordered eating among young adult college students both before (*B* = 0.60, *p* < 0.001) and after (*B* = 0.25, *p *< 0.001) accounting for self-perception as a hypothesized mediator (33).

Young adulthood is a period of life when emerging adults adopt lifestyle behaviors that very often lay the foundation for a certain health trajectory into later adulthood (35). There is a greater prevalence of disordered eating among young adults compared to middle age and older adults (36), which highlights the urgent need to establish a greater understanding of the link between ACEs and disordered eating among young adults. It is therefore essential to identify mediating mechanisms that attenuate the link between ACEs and disordered eating and could eventually be targeted to prevent or alleviate disordered eating among young adults with high ACEs.

Mindfulness is a hypothesized mediator of special consideration, as past study findings suggest that mindfulness is inversely related to ACEs (37–42) and disordered eating (43–46). Mindfulness involves the self-regulation of attention and responses related to internal (thoughts, feelings) and external (bodily senses, environment) stimuli (47). Intervention studies using mindfulness-based approaches have been shown to reduce the risk of poor mental health among individuals with high ACEs (48) and disordered eating symptoms (49–53). Mindfulness has been shown to attenuate the positive association that a high level of ACEs have with unhealthy behaviors like alcohol misuse (39) and domestic violence (38), poor health outcomes such as psychopathological symptoms (41) and depression (40), and health-related quality of life (37).

A recent study revealed how mindfulness moderated a positive link between depression and disordered eating by mitigating the effect of depression (54). A separate study highlighted how mindfulness was inversely related to both anxiety and disordered eating (55). These findings portray how mindfulness can mitigate the severity of disordered eating despite the presence of problematic circumstances that are linked to increased disordered eating. Additional outcomes from mediation studies have indicated that body dissatisfaction (56), perceived distress (57), anxiety (58, 59), depression (31, 59, 60), and emotion regulation (61–63) mediated the link between ACEs and disordered eating. Some mediators (i.e., perceived distress) were positively related to disordered eating, while others (i.e., emotion regulation) were inversely related to disordered eating.

Despite indications of mindfulness being inversely related to both ACEs and disordered eating, no known studies have explored whether mindfulness mediates this relationship. In being inversely related to ACEs and disordered eating, it is possible that mindfulness could play a mediating role by attenuating the positive relationship between ACEs and disordered eating in adulthood. Therefore, this critical knowledge gap needs to be addressed by determining whether mindfulness plays a mediating role within the relationship between ACEs and disordered eating.

### 1.3. Research hypotheses

The aims of this research were to address two primary knowledge gaps concerning ACEs and disordered eating among young adults using a cross-sectional observational study. First, research is scant regarding the link between ACEs and disordered eating among young adults who were not sampled from a university population. It is important to expand the knowledge base concerning how ACEs are related to disordered eating among young adults in the general population, as these individuals are in the process of developing and adopting health behaviors that could be sustained throughout their lives. Second, it is critical to identify mediators of the relationship between ACEs and disordered eating that can be tested in future intervention studies. The identification of mindfulness as a significant mediator of the relationship between ACEs and disordered eating would provide evidence that could be utilized for designing an intervention to increase mindfulness that can help address disordered eating among individuals with high ACEs. These study aims informed our two research hypotheses that (1) a positive relationship exists between ACEs and disordered eating among young adults, and (2) mindfulness mediates the relationship between ACEs and disordered eating among young adults.

## 2. Materials and methods

### 2.1. Participant recruitment

Study researchers implemented a quantitative, cross-sectional research approach to determine whether mindfulness mediates the link between ACEs and disordered eating among young adults in the U.S. A nationwide sample of 150 young adults in the U.S. were recruited to complete an online Qualtrics survey between May 3rd and May 8th, 2022 using CloudResearch from Amazon Mechanical Turk (MTurk) (64). MTurk is an online crowdsourcing platform offering an array of data collection features (65). A target sample size of 150 was the goal, as this exceeded the estimated sample size of 45 that was required to adequately power a cross-sectional study using multiple linear regression to conduct a two-tailed multiple linear regression analysis with five predictors, an anticipated effect size of 0.30 (31), an error probability of 0.05, and a power of 0.95 to detect a true positive (66). Data collected via MTurk has been found to be as reliable as data collected using more traditional methods (67), while also having the advantage of producing participant samples that are more diverse than other internet-recruited or college student samples (68, 69).

To be eligible for participation in the study, participants were required to be U.S. residents between the ages of 18 and 26 years who had never been clinically diagnosed with an eating disorder. Of the 150 survey responses, six respondents were excluded from the study due to the detection of three duplicate IP addresses, which resulted in a final study sample of 144 participants with no missing data. Three attention-check questions were used during data collection to promote data quality. One question was integrated into each of the ACEs, mindfulness, and disordered eating surveys. The attention-check question instructed respondents to select a certain answer. Data derived from respondents who did not select a correct answer for any attention-check question were excluded. The data collection process was completely anonymous. Participants received $2.00 for completing the 15-minute survey. Ethics approval was provided by the Institutional Review Board (IRB) of Arizona State University (STUDY00015736). All participants provided informed consent before joining the study.

### 2.2. Data collection measures

A 59-item online Qualtrics survey was used to obtain data on self-reported participant outcomes for ACEs, mindfulness, disordered eating, and personal characteristics.

ACEs were measured using the 11 items included within the Centers for Disease Control and Prevention’s 2020 Behavioral Risk Factor Surveillance System Survey (BRFSS) Questionnaire (70). The BRFSS items for ACEs include questions related to the following: fellow household members during childhood who experienced serious mental health problems, substance abuse, interpersonal violence, incarceration, and/or divorce; along with personal childhood experiences involving verbal, physical, and/or sexual abuse. In the BRFSS format, respondents are asked to indicate “Yes”, “No”, “Don't know/Not Sure”, or “Refused” for each item. For this study, respondents were given the options of “Yes” or “No” to preclude missing data. A psychometric evaluation of the ACEs measure used in the BRFSS survey have yielded results which indicated that the ACEs measure maintained an acceptable level of reliability that passed the threshold of 0.65 for multidimensional measures (*ω* = 0.906) (71).

Mindfulness was measured with the widely used 15-item Five-Facet Mindfulness Questionnaire (FFMQ) (72). The FFMQ items include statements covering five facets of mindfulness, which include: observing, describing, acting with awareness, non-judging of inner experience, and non-reactivity to inner experience. The FFMQ is answered on a five-point Likert scale, which ranges from “Never or very rarely true” to “Very often or always true”. The original FFMQ contains 39 items, so the 15-item FFMQ was developed and is used to minimize respondent burden since the two versions share consistent factor structures and strong correlations between total facet scores (73). Psychometric evaluations of the FFMQ have found that the, across the five facets of mindfulness, the FFMQ sustained an acceptable level of reliability for observing (*ω* = 0.699), describing (*ω* = 0.0.726), acting with awareness (*ω* = 0.771), non-judging (*ω* = 0.777), and non-reactivity (*ω* = 0.688) (74).

Disordered eating was measured using the 28-item Eating Disorder Examination Questionnaire (EDE-Q) (22). The EDE-Q contains the following four subscales of disordered eating behaviors: restraint, eating concern, shape concern, and weight concern. Altogether, the four disordered eating subscales can be combined to create the general outcome of global disordered eating. Likert scales with varying point ranges are used in the EDE-Q to gauge either the frequency (e.g., No days, Every day) or intensity (e.g., Not at all, Markedly) of an outcome that contributes to a disordered eating subscale. Examinations of the test-retest reliability of the EDE-Q have concluded that the EDE-Q has strong measurement reliability for the subscales of restraint (*r* = 0.81), eating concern (*r* = 0.87), shape concern (*r* = 0.94), and weight concern (*r* = 0.92) (75).

Participant characteristics measured in the study included age in years (18–26), biological sex (female, male), race/ethnicity (American Indian or Alaska Native, Asian, Black, Hispanic/Latino, White), education (high school, some college, Bachelor's degree, Master's degree, Doctoral degree), and income (<$25,000; $25,000–$49,000; $50,000–$74,999; $75,000–$99,000; ≥$100,000). The full study survey in Qualtrics required that participants provide a response to each ACEs, mindfulness, disordered eating, and personal characteristics item before proceeding to the next item. This approach prevented instances of missing data from occurring throughout data collection.

### 2.3. Primary variables and covariates

ACEs were the primary predictor variable in this study. A continuous interval variable for ACEs was created by adding responses for each of the 11 ACEs items (no = 0, yes = 1) and then assigning each participant a total ACEs score (0–11). Separately, a dichotomous ACEs variable was created to categorize participants by whether they had experienced four or more ACEs (ACEs < 4 = 0, ACEs ≥ 4 = 1). A cutoff point of ≥4 was chosen because encountering four or more types of ACEs has been recognized as a widely accepted threshold for individuals being at an increased risk of poor health and adverse social outcomes in adulthood when compared to those with less than four ACEs (76). The continuous ACEs score was used to test differences in mindfulness and disordered eating across different levels of ACEs. Separately, the dichotomous ACEs score was used for descriptive purposes and to assess the association between having four or more ACEs and disordered eating.

Mindfulness was the hypothesized mediating variable in the link between ACEs and disordered eating. Scores for each FFMQ item ranged from one to five points with some items being reverse scored. A continuous variable was created for mindfulness by summing the total score for all 15 items and dividing the sum by 15 to assign each participant a mean mindfulness score (0–5). Disordered eating was the primary outcome variable in this study. Continuous variables were created for each of the disordered eating subscales (restraint, eating concern, weight concern, shape concern) by summing the item totals and dividing by the number of items to produce a mean subscale score. The four disordered eating subscale scores were then summed and divided by four to produce a continuous global disordered eating score. Covariates in this study included all participant characteristics. Age was zero-centered when coded as an interval variable. Sex was dichotomously coded as biological female or male. Race/ethnicity was treated as a categorical variable. Ordinal variables for both Education and Income were created, although Education was eventually excluded in statistical analyses due to multicollinearity resulting from a strong correlation with Income.

### 2.4. Statistical analyses

RStudio packages including “glm”, “rmediation”, and “stats” were utilized for analyzing the study data (77). Generalized Linear Models were used to perform bivariate and multivariable linear regression analyses to examine cross-sectional relationships between ACEs and disordered eating, ACEs and mindfulness, and mindfulness and disordered eating. Across all analyses, statistical significance was set at *p *< 0.05. Bivariate linear regression models analyzed the link between having ≥4 ACEs and global disordered eating scores. Multiple linear regression analyses were then used to assess the link between having ≥4 ACEs and global disordered eating while adjusting for the potential confounding influences of covariates (i.e., participant characteristics). Multiple linear regression analyses were also conducted to determine the association between ACEs score and mindfulness, ACEs score and global disordered eating, ACEs score and restraint, ACEs score and eating concern, ACEs score and weight concern, and ACEs score and shape concern. All multiple linear regression analyses statistically adjusted for the covariates of age, sex, race/ethnicity, and income.

Mediation analyses were then performed by computing the product-of-coefficients (*ab*) using the unstandardized beta coefficients and standard errors produced from the multivariable linear regression analyses to produce mediating effect size coefficients, standard errors, and 95% confidence intervals (CI) for each disordered eating outcome (78–80). In the mediation analyses, the *a*-path assessed the link between ACEs and mindfulness, the *b*-path assessed the link between mindfulness and disordered eating, the *c*-path assessed the link between ACEs and disordered eating, and the *c’*-path assessed the link between ACEs and disordered eating adjusted for mindfulness.

Five separate mediation analyses were conducted to model global disordered eating, restraint, eating concern, weight concern, and shape concern as the outcome variable. Coefficients and standard errors for paths *a* and *b* were used to compute *ab* for determining the extent to which mindfulness mediates the link between ACEs and global disordered eating along with the four disordered eating subscales of restraint, eating concern, weight concern, and shape concern. All mediation analyses adjusted for the covariates of age, sex, race/ethnicity, and income.

## 3. Results

### 3.1. Participant characteristics

This study included a participant sample composed of young adults between the ages of 18–26 (*n* = 144) who reside in the U.S. and have no previous clinical diagnosis of an eating disorder (Table 1). The mean average age of participants was 23.4 (SD = 2.4). Participants were mostly male (59%), White (66.7%), with an annual income of $25,000–$49,999 (29.9%). Across the study sample (*n* = 144), the mean average number of different types of ACEs that participants experienced in childhood were 2.80 (SD = 2.72). The mean mindfulness score among participants was 3.43 (SD = 0.48). For disordered eating, mean scores were 2.04 (SD = 1.44) for global disordered eating, 1.60 (SD = 1.68) for restraint, 2.56 (SD = 1.11) for eating concern, 1.97 (SD = 1.70) for weight concern, and 2.03 (SD = 1.72) for shape concern.

**Table 1:** Participant characteristics among U.S. young adults (*n* = 144).

Characteristics	Total (%)
Age (years)	*M* = 23.4, SD = 2.4^a^
Sex	
Female	59 (41)
Male	85 (59)
Race/Ethnicity	
American Indian/Alaska Native	2 (1.4)
Asian	9 (6.2)
Black	29 (20.1)
Hispanic/Latino	8 (5.6)
White	96 (66.7)
Income	
<$25,000	36 (25)
$25,000–$49,999	43 (29.9)
$50,000–$74,999	37 (25.7)
$75,000–$99,999	17 (11.8)
≥$100,000	11 (7.6)
Adverse childhood experiences	*M* = 2.80, SD = 2.72
<4	98 (68.1)
≥4	46 (31.9)
Mindfulness	*M* = 3.43, SD = 0.48
Disordered eating	
Global	*M* = 2.04, SD = 1.44
Restraint	*M* = 1.60, SD = 1.68
Eating concern	*M* = 2.56, SD = 1.11
Weight concern	*M* = 1.97, SD = 1.70
Shape concern	*M* = 2.03, SD = 1.72

^a^ *M*, mean; SD, standard deviation.

By descending order, the most prevalent individual ACEs (Table 2) among young adults (*n* = 144) in this study sample included: verbal abuse by an adult in the home (43.8%); parents or guardians separating or getting divorced (41.7%); living with someone who had a mental illness (34.7%); living with someone who was a problem drinker (33.3%); being a victim of physical abuse (28.5%); witnessing physical violence among adults in the home (20.8%); living with someone who abused drugs (19.4%); living with someone who served time in prison or jail (19.4%); and having someone at least 5 years older than them touch them sexually (15.3%), request sexual touching (12.5%), and/or force them to have sex (10.4%).

**Table 2:** Prevalence of adverse childhood experience among U.S. young adults (*n* = 144).

Adverse childhood experiences	No (%)	Yes (%)
Lived with someone who had a mental illness.	94 (65.3)	50 (34.7)
Lived with someone who was a problem drinker.	96 (66.7)	48 (33.3)
Lived with someone who abused drugs.	116 (80.6)	28 (19.4)
Lived with someone who served time in prison or jail.	116 (80.6)	28 (19.4)
Parents or guardians were separated or divorced.	84 (58.3)	60 (41.7)
Witnessed physical violence among adults in the home.	114 (79.2)	30 (20.8)
Was a victim of physical abuse.	103 (71.5)	41 (28.5)
Was verbally abused by an adult in the home.	81 (56.2)	63 (43.8)
Was touched sexually by someone at least 5 years older.	122 (84.7)	22 (15.3)
Someone at least 5 years older requested sexual touching.	126 (87.5)	18 (12.5)
Was forced to have sex with someone at least 5 years older.	129 (89.6)	15 (10.4)

### 3.2. Univariate and multivariable regression for ≥4 ACEs and disordered eating

A majority of participants encountered fewer than four ACEs (68.1%). Univariate regression analyses (Table 3) yielded results suggesting a significant association between having four or more ACEs and disordered eating status (*B* = 1.03, SE = 0.24; *p* < 0.001). Findings from multivariable regression analyses adjusted for covariates supported the univariate results by indicating a significant positive association between having four or more ACEs and global disordered eating scores (*B* = 0.97, SE = 0.25; *p *< 0.001).

**Table 3:** The relationship between encountering four-or-more adverse childhood experiences and global disordered eating scores among U.S. young adults (*n* = 144).

Model	Statistic	*p*-value
Univariate^a^	*B* = 1.03, SE = 0.24^c^	*p <* 0.001
Multivariable^b^	*B* = 0.97, SE = 0.25^c^	*p *< 0.001

^a^ Univariate analysis included linear regression using a generalized linear model.

^b^ Multivariable analysis included linear regression using a generalized linear model adjusted for age, sex, race/ethnicity, and income.

^c^ B, unstandardized beta coefficient; SE, standard error.

### 3.3. Mediation via multivariable linear regression and product-of-coefficients

**Figure 1:**
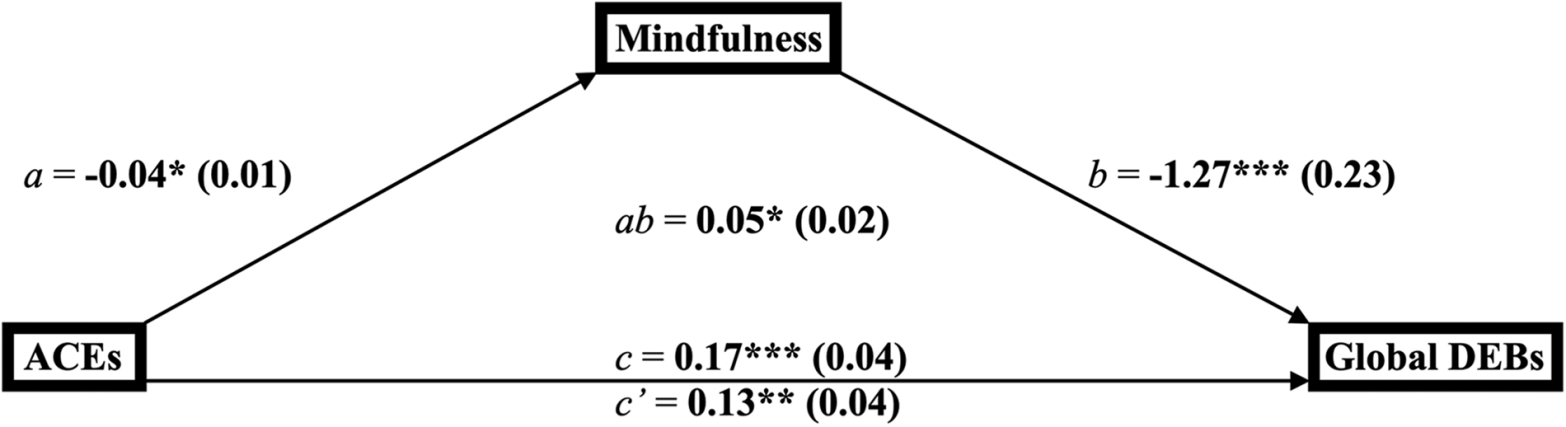
Mediation diagram for the link between adverse childhood experiences, mindfulness, and global disordered eating behaviors among young adults (*n* = 144). ^a^*p < 0.05, **p < 0.01, ***p < 0.001. ^b^ab = product-of-coefficients mediation statistic. ^c^a, b, c = direct effect paths; *c’* = indirect effect path. ^d^ACES, adverse childhood experiences; DEBs, disordered eating behaviors.

The conceptualized mediation diagram for the relationship between ACEs, mindfulness, and global disordered eating (Figure 1) depicts ACEs as the predictor, mindfulness as the mediator, and global disordered eating as the outcome.

#### 3.3.1. Mediation model for ACEs, mindfulness, and global disordered eating

Findings from the multivariable regression analyses (Table 4) suggest that ACEs were inversely related to mindfulness (*B* = −0.04, SE = 0.01; 95% CI = −0.07, −0.01; *p *= 0.01), mindfulness was inversely related to global disordered eating (*B* = −1.27, SE = 0.23; 95% CI = −1.74, −0.80; *p *< 0.0001), ACEs were positively related to global disordered eating (*B* = 0.17, SE = 0.04; 95% CI = 0.09, 0.26; *p *= 0.0001), and ACEs remained positively related to global disordered eating after adjusting for mindfulness (*B* = 0.13, SE = 0.04; 95% CI = 0.05, 0.21; *p *= 0.002). The product-of-coefficients indicate the link between ACEs and global disordered eating was mediated by mindfulness (*B* = 0.05, SE = 0.02; 95% CI = 0.01, 0.09; *p *< 0.05).

**Table 4:** Mediation analyses for the link between adverse childhood experiences, mindfulness, and disordered eating behaviors among U.S. young adults (*n* = 144).

	Path	*B*, SE (95% CI)^a^	*p*-value
Global DEBs^a^			
*a*-path	ACEs → Mindfulness	−0.04, 0.01 (−0.07, −0.01)	*p *= 0.01
*b*-path	Mindfulness → Global DEBs	−1.27, 0.23 (−1.74, −0.80)	*p *< 0.0001
*c*-path	ACEs → Global DEBs	0.17, 0.04 (0.09, 0.26)	*p *= 0.0001
*c’*-path	ACEs → Global DEBs	0.13, 0.04 (0.05, 0.21)	*p *= 0.002
*Mediating effect of Mindfulness (ab)* ^b^		0.05, 0.02 (0.01, 0.09)	*p *< 0.05
Restraint			
*a*-path	ACEs → Mindfulness	−0.04, 0.01 (−0.07, −0.01)	*p *< 0.05
*b*-path	Mindfulness → Restraint	−1.09, 0.28 (−1.65, −0.53)	*p *= 0.0002
*c*-path	ACEs → Restraint	0.16, 0.05 (0.06, 0.26)	*p *= 0.002
*c’*-path	ACEs → Restraint	0.13, 0.05 (0.03, 0.22)	*p *= 0.013
*Mediating effect of Mindfulness (ab)* ^b^		0.04, 0.02 (0.02, 0.08)	*p *< 0.05
Eating Concern			
*a*-path	ACEs → Mindfulness	−0.04, 0.01 (−0.07, −0.01)	*p *< 0.05
*b*-path	Mindfulness → Eating Concern	−0.96, 0.19 (−1.33, −0.59)	*p *< 0.0001
*c*-path	ACEs → Eating Concern	0.12, 0.03 (0.06, 0.20)	*p *= 0.0002
*c’*-path	ACEs → Eating Concern	0.10, 0.03 (0.03, 0.16)	*p *= 0.003
*Mediating effect of Mindfulness (ab)* ^b^		0.04, 0.01 (0.02, 0.07)	*p *< 0.05
Weight Concern			
*a*-path	ACEs → Mindfulness	−0.04, 0.01 (−0.07, −0.01)	*p *< 0.05
*b*-path	Mindfulness → Weight Concern	−1.48, 0.28 (−2.04, −0.93)	*p *< 0.0001
*c*-path	ACEs → Weight Concern	0.21, S0.05 (0.11, 0.31)	*p *< 0.0001
*c’*-path	ACEs → Weight Concern	0.17, 0.05 (0.07, 0.26)	*p *= 0.0009
*Mediating effect of Mindfulness (ab)* ^b^		0.06, 0.02 (0.03, 0.10)	*p *< 0.05
Shape Concern			
*a*-path	ACEs → Mindfulness	−0.04, 0.01 (−0.07, −0.01)	*p *< 0.05
*b*-path	Mindfulness → Shape Concern	−1.56, 0.28 (−2.11, −1.00)	*p *< 0.0001
*c*-path	ACEs → Shape Concern	0.18, 0.05 (0.08, 0.28)	*p *= 0.0008
*c’*-path	ACEs → Shape Concern	0.13, 0.05 (0.03, 0.22)	*p *= 0.01
*Mediating effect of Mindfulness (ab)* ^b^		0.06, 0.02 (0.03, 0.10)	*p *< 0.05

^a^ ACEs, adverse childhood experiences; B, unstandardized beta coefficient; CI, confidence interval; DEBs, disordered eating behaviors; SE, standard error.

^b^ Mediation analyses computed with product-of-coefficients using unstandardized beta coefficients derived from multivariable generalized linear models adjusted for age, sex, race/ethnicity, and income.

#### 3.3.2. Mediation models for ACEs, mindfulness, and the disordered eating subscales

**Figure 2:**
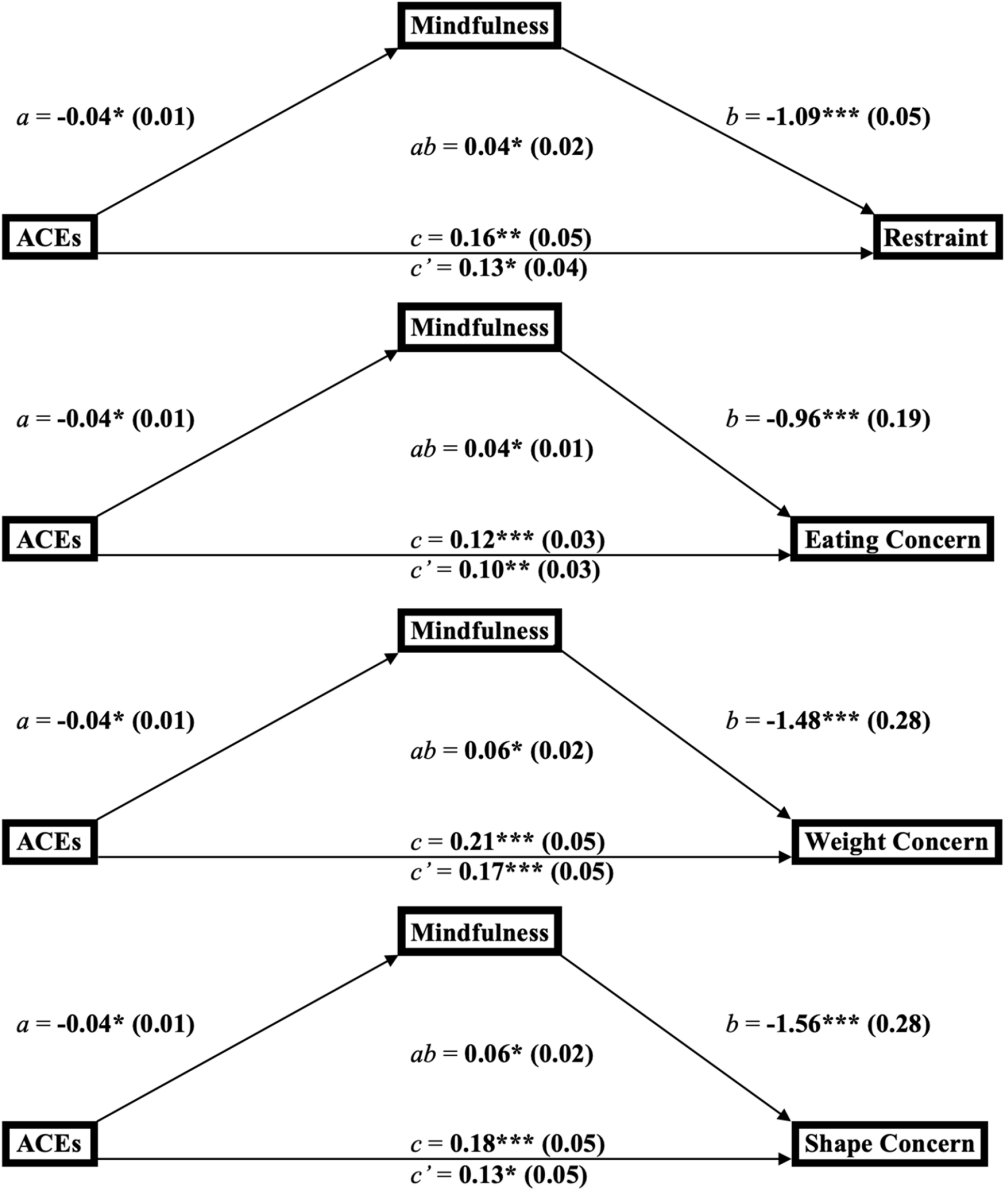
Mediation diagrams for the links between adverse childhood experiences, mindfulness, and the subscales of disordered eating behaviors among young adults (*n* = 144). ^a^*p < 0.05, **p < 0.01, ***p < 0.001. ^b^ab = product-of-coefficients mediation statistic. ^C^a, b, c = direct effect paths; *c’* = indirect effect path. ^d^ACES, adverse childhood experiences.

The conceptualized mediation models for the relationship between ACEs, mindfulness, and either restraint, eating concern, weight concern, or shape concern (Figure 2) depict ACEs as the predictor, mindfulness as the mediator, and each of the four aforementioned disordered eating subscales as the outcome.

#### 3.3.3. Mediation model for ACEs, mindfulness, and restraint

In the mediation analysis with restraint as the outcome, findings suggest ACEs were inversely related to mindfulness (*B* = −0.04, SE = 0.01; 95% CI = −0.07, −0.01; *p *< 0.05), mindfulness was inversely related to restraint (*B* = −1.09, SE = 0.28; 95% CI = −1.65, −0.53; *p *= 0.0002), ACEs were positively related to restraint (*B* = 0.16, SE = 0.05; 95% CI = 0.06, 0.26; *p *= 0.002), and ACEs remained positively related to restraint after adjusting for mindfulness (*B* = 0.13, SE = 0.05; 95% CI = 0.03, 0.22; *p *= 0.013). The product-of-coefficients indicate the link between ACEs and restraint was mediated by mindfulness (*B* = 0.04, SE = 0.02; 95% CI = 0.02, 0.08; *p *< 0.05).

#### 3.3.4. Mediation model for ACEs, mindfulness, and eating concern

The mediation analysis modeling eating concern as the outcome produced results indicating that ACEs were inversely related to mindfulness (*B* = −0.04, SE = 0.01; 95% CI = −0.07, −0.01; *p *< 0.05), mindfulness was inversely related to eating concern (*B* = −0.96, SE = 0.19; 95% CI = −1.33, −0.59; *p *< 0.0001), ACEs were positively related to eating concern (*B* = 0.12, SE = 0.03; 95% CI = 0.06, 0.20; *p *= 0.0002), and ACEs remained positively related to eating concern after adjusting for mindfulness (*B* = 0.10, SE = 0.03; 95% CI = 0.03, 0.16; *p *= 0.003). The product-of-coefficients indicated the link between ACEs and eating concern was mediated by mindfulness (*B* = 0.04, SE = 0.01; 95% CI = 0.02, 0.07; *p *< 0.05).

#### 3.3.5. Mediation model for ACEs, mindfulness, and weight concern

A mediation analysis with weight concern as the outcome produced results indicating ACEs were inversely related to mindfulness (*B* = −0.04, SE = 0.01; 95% CI = −0.07, −0.01; *p *< 0.05), mindfulness was inversely related to weight concern (*B* = −1.48, SE = 0.28; 95% CI = −2.04, −0.93; *p *< 0.0001), ACEs were positively related to weight concern (*B* = 0.21, SE = 0.05; 95% CI = 0.11, 0.31; *p *< 0.0001), and ACEs remained positively related to weight concern after adjusting for mindfulness (*B* = 0.17, SE = 0.05; 95% CI = 0.07, 0.26; *p *= 0.0009). The product-of-coefficients indicated the link between ACEs and weight concern was mediated by mindfulness (*B* = 0.06, SE = 0.02; 95% CI = 0.03, 0.10; *p *< 0.05).

#### 3.3.6. Mediation model for ACEs, mindfulness, and shape concern

Lastly, the mediation analysis modeling shape concern as the outcome produced results indicating ACEs were inversely related to mindfulness (*B* = −0.04, SE = 0.01; 95% CI = −0.07, −0.01; *p *< 0.05), mindfulness was inversely related to shape concern (*B* = −1.56, SE = 0.28; 95% CI = −2.11, −1.00; *p *< 0.0001), ACEs were positively related to shape concern (*B* = 0.18, SE = 0.05; 95% CI = 0.08, 0.28; *p *= 0.0008), and ACEs remained positively related to shape concern after adjusting for mindfulness (*B* = 0.13, SE = 0.05; 95% CI = 0.03, 0.22; *p *= 0.01). The product-of-coefficients indicated the link between ACEs and shape concern was mediated by mindfulness (*B* = 0.06, SE = 0.02; 95% CI = 0.03, 0.10; *p *< 0.05).

## 4. Discussion

### 4.1. The association between ACEs and disordered eating

Outcomes from this study further confirmed the findings of existing studies that suggested ACEs may contribute to disordered eating behaviors among young adults. Findings from this study supported results from past research that emphasized the positive association between various types of ACEs [e.g., household dysfunction (20), neglect (31, 32, 34), and abuse (32–34)] and disordered eating. Across all participants, high ACEs scores were related to an increased severity of disordered eating. Furthermore, disordered eating was more severe among young adults with four or more ACEs when compared to those with fewer than four ACEs. These results are supported by previous studies among non-college student young adults that reported household dysfunction (20), abuse (20), and neglect (34) during childhood as potent predictors of displaying disordered eating symptoms later in life. Findings among young adult college students further support results in the present study by highlighting the association between physical abuse (31, 32), emotional abuse (31–33), sexual abuse (31, 32), and neglect (31, 32) during childhood and disordered eating in adulthood. The increased risk of habitual disordered eating behaviors among young adults with high ACEs raises concerns about the health trajectories of these individuals across their life course.

### 4.2. Links between ACEs and restraint, eating concern, weight concern, or shape concern

ACEs were positively associated with the disordered eating subscales of restraint, eating concern, weight concern, and shape concern. The positive link between ACEs and restraint that was detected in this study is discordant with non-significant findings from the only other known study to have analyzed the link between ACEs and restraint among a non-clinical sample of adults (81). Although, these findings could be discordant due to alternative explanations relating to differences in the research methodology, as Wingenfeld et al. recruited an all-female study sample and did not statistically control for the potential confounding variables of age, race/ethnicity, and income. Given the conflicting evidence, the present study offers a new perspective for the risk that ACEs pose to food avoidance behaviors. ACEs were also positively linked to eating concern, which has been posited as an antecedent to binge eating (82), a trait characterized by eating a large amount of food in a short period of time (83).

Moreover, the positive link detected between ACEs and weight concern highlights an increased risk for other eating disorders including binge eating, bulimia, and anorexia nervosa, as these three eating disorders have been linked to the presence of weight concern (84, 85). Findings detailing the relationships between ACEs and either eating concern or shape concern are supported by recent research emphasizing how individuals with clinically diagnosed eating disorders have significantly higher ACEs than those with no eating disorder diagnosis (86). Moreover, the positive link between ACEs and shape concern supports findings from past studies underscoring the connection between ACEs, body dissatisfaction (87), and body dysmorphia (88). The evidence is consistent across all four subscales of disordered eating in showing the multifarious risk that ACEs pose to the development of these unhealthy behaviors.

### 4.3. Mindfulness mediating the association between ACEs and disordered eating

Identifying mindfulness as a significant mediator in the relationship between ACEs and disordered eating was key to answering to the research hypotheses in this study. In five separate statistical models, mindfulness was shown to significantly mediate the link between ACEs and global disordered eating, restraint, eating concern, weight concern, and shape concern. These results raise important considerations concerning the underlying mechanisms that would contribute to mindfulness being a temporal mediator had this been a longitudinal study that tracked the effect of ACEs on mindfulness and the effect of mindfulness on disordered eating over time. Mindfulness involves the self-regulation of personal responses to internal and external stimuli (47), so emotion regulation could be a possible mechanism of mindfulness as a mediator since emotion regulation has been shown to blunt the harmful impact of ACEs on disordered eating.

The significant relationship that existed between ACEs and mindfulness should be perceived with caution, as this cross-sectional study could not determine cause-and-effect results and the coefficient detected for this relationship was small, which could both limit the legitimacy of mindfulness being a treatment target for interventions. Nonetheless, these findings help to tie together evidence from past studies that established links between ACEs and mindfulness (37) and between mindfulness and disordered eating (43) by introducing mindfulness as a potential mediator within the temporal relationship between ACEs and disordered eating. The discoveries made in this study fill an important knowledge gap concerning the relationship between ACEs and disordered eating, as the role of mindfulness in this context was previously unknown. Given that mindfulness is a modifiable personality trait that can be changed over time (89, 90), the evidence from the present study provided a glimpse for whether a mindfulness intervention could be used to alleviate disordered eating among adults with high ACEs.

### 4.4. Past and future mindfulness interventions

Past studies have successfully implemented a mindfulness intervention that successfully reduced disordered eating symptoms of restraint (50, 52), eating concern (50, 51, 53), weight concern (49, 50), and shape concern (49, 50) among samples of adolescents (49), adult females (52), adults who were overweight or obese (51, 53), and adults with a clinically diagnosed eating disorder (50). Preventing ACEs from contributing to the development and exacerbation of disordered eating throughout adulthood is essential for improving public health outcomes, and mindfulness could play an impactful role in this process. Parenting techniques that incorporate mindfulness through specific parent-child interactions have been shown to supplement traditional approaches to promoting healthy behaviors among children (91). Advocacy has been increasing to also teach mindfulness practices to children in school settings (92), as improved mindfulness in grade school students has been connected to enhanced emotion regulation (93), a more positive outlook (94), increased prosocial behavior (93), better academic outcomes (95), and greater life satisfaction (94). Therefore, future research is needed to identify the most effective mindfulness practices for children and test the extent to which promoting these mindfulness approaches among families and schools can alleviate and prevent disordered eating among young adults with high ACEs.

### 4.5. Strengths and limitations

This research contained multiple strengths. First, study findings expanded the knowledge base concerning the relationship between ACEs and disordered eating among young adults in the U.S., as most previous studies examining ACEs and disordered eating among this population exclusively sampled college students, while this study was open to all young adults. Second, the cross-sectional mediation models used in this study yielded unique information detailing how mindfulness mediated the link between ACEs and disordered eating, which could be used to rationalize further exploration of whether this significant relationship would be maintained in a longitudinal study. Third, the young adult sample recruited for this study was not exclusively composed of college students, which enhanced the generalizability of these study findings to other young adults in the U.S.

There were also several limitations in this study. First, this study was cross-sectional, which prohibited the assigning of causal inference to the hypothesized effect of ACEs on mindfulness and disordered eating. Temporality in the relationship between ACEs, mindfulness and disordered eating was assumed since data for ACEs are restricted to the ages of 0–17 while data for mindfulness and disordered eating were based on the present day at the time of data collection, but longitudinal data is needed to best determine the truth regarding the relationships that were explored in this study. Second, participants in this study were not randomly selected. A convenience sample of young adults was recruited for this study, which introduced self-selection bias. Third, data for primary variables were obtained from self-report measures, which are limited in their accuracy due to subjectivity, recall bias, and social desirability bias. For example, the 11-item ACEs measure can only subjectively detect occurrences that occurred in childhood through honest answers to closed-ended questions and cannot objectively pinpoint more in-depth family processes that took place. Three attention-check questions were integrated into the study survey that simply asked participants to select a specific answer, which helped to strengthen the validity of survey responses since it could be confirmed that respondents were actually reading the questions before providing their responses Fourth, two-thirds of the participants in the study sample were White, which limits the extent to which these findings can be generalized to racial and ethnic minority groups. Potential implications of this limitation include ongoing uncertainty concerning how the relationship between ACEs and disordered eating differs across racial and ethnic groups. Fifth, the product-of-coefficients approach to estimating the mediating effects in this study produced results that only indicated whether the findings were significant at the 0.05 level, and did not provide a specific *p*-value despite providing specific effect size coefficients, standard errors, and 95% confidence intervals. For this reason, the information pertaining to *p*-values in the mediation analyses are limited to the findings either being less-than 0.05 or greater-than 0.05. This limitation did not prevent the hypotheses from being answered, as the resulting effect size coefficients, standard errors, 95% confidence intervals, and general (yet not specific) *p*-values provided sufficient information to address the primary research questions. Lastly, while the coefficients for the mediating effect of mindfulness were statistically significant, the effect sizes were small which raise concerns about whether mindfulness plays a clinically significant role within the relationship between ACEs and disordered eating. For this reason, additional studies are needed to confirm whether the small yet significant mediating effect of mindfulness that has been detected among non-clinical sample would still be significant among a clinical sample of young adults with high ACEs.

## 5. Conclusions

Young adults with high ACEs are at an increased risk of disordered eating. Evidence suggested that mindfulness played an influential role in the relationship between ACEs and disordered eating, but longitudinal research is needed to better estimate the interplay of mindfulness within the relationship between ACEs and disordered eating among young adults. Additional studies among diverse samples of young adults are needed to strengthen the evidence for the relationship between ACEs, mindfulness, and disordered eating by producing findings that are generalizable to wider variety of people groups. Future intervention studies among young adults with high ACEs are needed to evaluate whether mindfulness practices can meaningfully decrease and prevent disordered eating. Such public health efforts could generate effective disease prevention approaches to promote and ensure health equity among vulnerable populations.

## Data Availability

The raw data supporting the conclusions of this article will be made available by the authors, without undue reservation.
